# Manual restricted kinematic alignment technique restores postoperative limb alignment in severe knee deformities

**DOI:** 10.1038/s41598-025-13195-w

**Published:** 2025-07-30

**Authors:** Masahiro Ishikawa, Masaaki Ishikawa, Hideaki Nagashima, Takafumi Hiranaka

**Affiliations:** 1 Department of Orthopedic Surgery, Nagahama Red Cross Hospital, Miyamae, Nagahama, Shiga 526-0053 Japan; 2 Department of Orthopedic Surgery, Fukui Red Cross Hospital, Tukimi 2-4-1, Fukui, 918-8501 Japan; 3 Department of Otolaryngology, Head and Neck Surgery, Hyogo Prefectural Amagasaki General Medical Center, 2-17-77 Higashinaniwachou, Amagasaki, Hyogo Prefecture 660-8550 Japan; 4 Department of Orthopedic Surgery and Joint Surgery Centre, Takatsuki General Hospital, 1-3-13 Kosobe-Cho, Takatsuki, 569-1192 Osaka Japan

**Keywords:** Total knee arthroplasty, Restricted kinematically aligned TKA, Kinematic alignment, Hip–knee–ankle angle, Diseases, Medical research, Materials science

## Abstract

Restricted kinematically aligned total knee arthroplasty (rKA-TKA) for severe deformity in the preoperative hip–knee–ankle angle (HKAA) has gained considerable interest. However, the widespread adoption of rKA-TKA has been limited by its requirement for expensive equipment such as navigation and robotic surgery systems. In this study, we developed manual rKA-TKA with modified soft tissue-respecting technique and investigated its surgical effects on postoperative HKAA. To achieve this, we examined factors affecting postoperative HKAA. Subsequently, the safe zone-related cut-off values of preoperative HKAA were calculated using the receiver operating characteristic curve, and postoperative HKAA was predicted using a linear regression model (LRM) and generalized additive model (GAM). Preoperative HKAA was identified as a factor influencing postoperative HKAA. The cut-off values of preoperative HKAA were −14 and −15° when the safe zones were defined as ± 1–3 and ± 4–5°, respectively. The GAM was more accurate in predicting the postoperative HKAA than the LRM. Additionally, the GAM showed a potential of falling within ± 5° of the postoperative HKAA, even in patients with preoperative HKAA ≤ − 19°. These findings suggest that manual rKA-TKA can be effective even for patients with severe deformities, providing an accessible alternative to conventional TKA for surgeons at resource-limited institutions.

**Supplementary Information:**

The online version contains supplementary material available at 10.1038/s41598-025-13195-w.

## Introduction

Mechanically aligned total knee arthroplasty (MA-TKA) aims for neutral alignment, defined as a hip–knee–ankle angle (HKAA) of 0°, and has been performed as a representative TKA^1,2^. In MA-TKA, a postoperative HKAA falling within a safe range of ± 3° is associated with better implant survival^3–5^. While MA-TKA has excellent long-term survivorship, 20% of patients are reportedly dissatisfied with residual symptoms after MA-TKA^6,7^. One plausible explanation for these results may be the endeavor to achieve neutral alignment in all patients after MA-TKA, irrespective of the preoperative HKAA, which can be recognized as the “one size fits all” approach. Previous studies indicated the rarity of neutral alignment even in healthy populations^8^ and reported that constitutional varus alignment was prevalent, particularly in the Asian population^9,10^. Consequently, the concept of unrestricted kinematically aligned TKA (urKA-TKA) was developed^11,12^. The crucial concept of urKA-TKA is based on reproducing the native or pre-arthritic alignment in each patient, which is considered a “personalized” or “individualized” approach^13,14^. Notably, urKA-TKA reproduces the native articular surface of the femur, which plays a central role in knee motion^15^. There are two types of tibial resection in urKA-TKA—namely, the calipered technique^11^ and the soft tissue-respecting technique (STRT)^16,17^.

Owing to the reproducibility of personalized pre-arthritic limb alignment in each patient, urKA-TKA has become attractive to surgeons. However, urKA-TKA aims to restore the pre-arthritic alignment without limiting the bone resection angles^18,19^. As postoperative HKAA is theoretically dependent on preoperative HKAA, some surgeons may hesitate to perform urKA-TKA in patients with severe degrees of preoperative HKAA because of the possibility of severe deformity remaining in the postoperative HKAA. Currently, as no systematic reviews or meta-analyses of the KA-TKA-related safe zone for postoperative HKAA have been conducted^20^, surgeons perform urKA-TKA with the aim of falling into the postoperative HKAA within the MA-TKA-derived safe zone. Previously, we investigated surgical effects on the postoperative HKAA in Japanese patients who underwent urKA-TKA with the modified STRT^21^. By using a nonlinear regression model, our previous study revealed that patients with preoperative HKAA ≤-19°, -15°, and − 12° were less likely to have postoperative HKAA falling within ± 5°, ± 4°, and ± 3° of the postoperative HKAA, respectively, in urKA-TKA with the modified STRT. Based on these results, surgeons may hesitate to perform urKA-TKA with the modified STRT in severe deformity patients with preoperative HKAA ≤-19° when the safe range is defined as ± 5°.

**Fig. 1:**
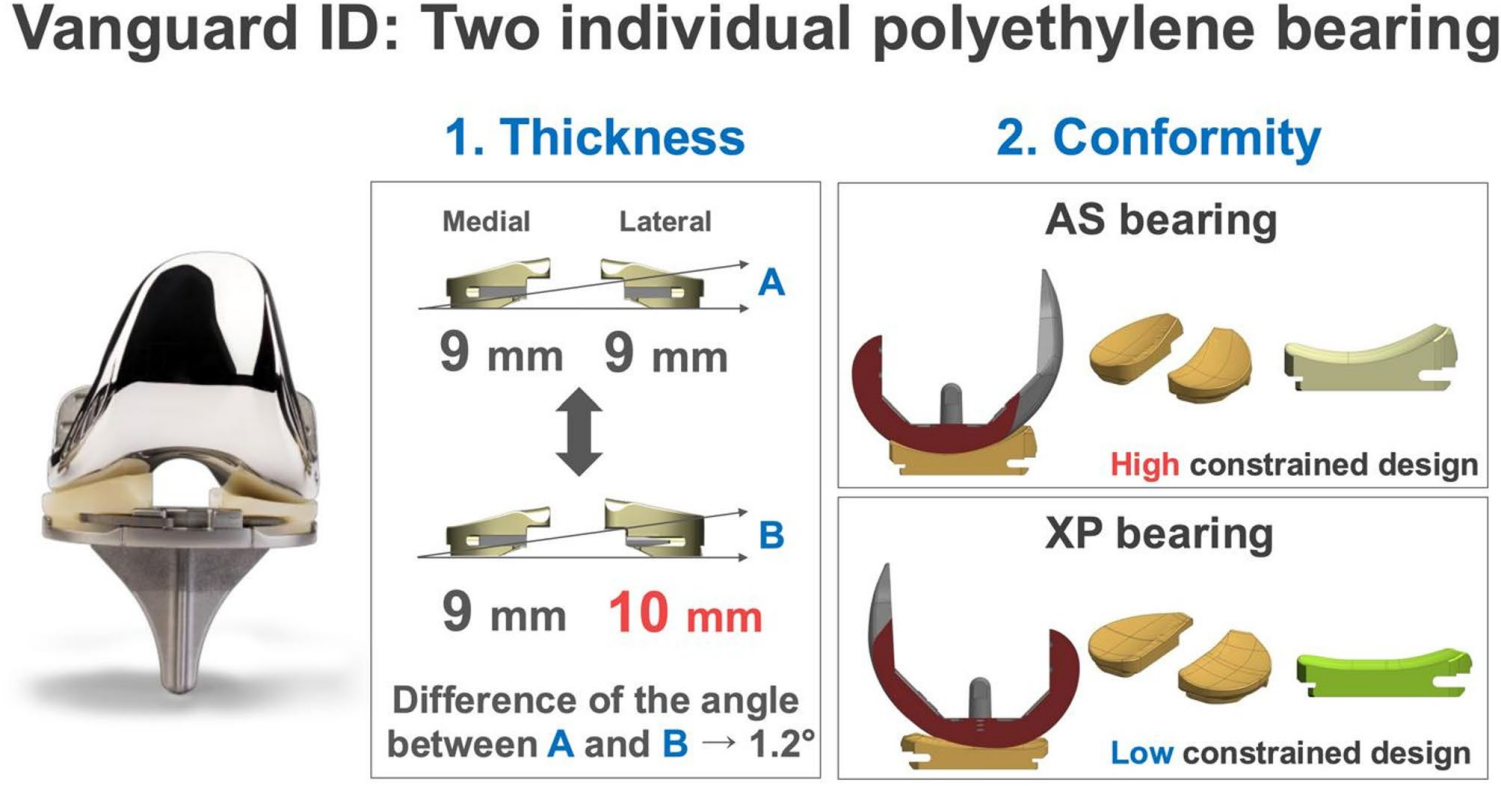
Characteristic of Vanguard ID (CR implant). *Thickness*: Vanguard ID allows for changes in thickness independently. Every 1-mm difference in the bearing results in 1.2° of joint-line inclination (e.g., 9-mm medial bearing and 10-mm lateral bearing result in 1.2° of joint-line inclination). *Constraints*: Vanguard ID also allows for changes in conformity independently. Two types of bearing conformity exist—namely, the anterior stabilized (AS) bearing with high constrained design (tibia/femoral coronal conformity ratio: 1:1) and the bicruciate-preserving (XP) bearing with low constrained design (tibia/femoral coronal conformity ratio: 1.25:1). In all knees, an AS bearing is used on the medial side, whereas an XP bearing is used on the lateral side. *AS* anterior stabilized, *XP* bicruciate-preserving, *CR* cruciate-retaining.

Restricted kinematically aligned TKA (rKA-TKA) is based on the concept of restricting the bone resection angle of the femur and/or tibia within the safe zone^22–24^. Recently, rKA-TKA for patients with severe preoperative HKAA has gained much interest, especially in Asian populations. The rKA-TKA can be performed within the MA-KTA-derived safe zone and is therefore a promising approach for patients with severe preoperative HKAA. A previous report showed that rKA-TKA could restore the kinematics of the native knee, prevent excessive coronal limb alignment, and provide the same or slightly better patient-reported outcome measures than MA-TKA without increasing the risk of midterm implant failure^25^. Nevertheless, rKA-TKA has some limitations with respect to the surgical approach. Computer-assisted surgical navigation is indispensable because it requires a precise bone resection angle^14,26,27^. Two previous studies estimated an additional cost of approximately 1,500–3,000 US dollar per patient when applying advanced techniques, compared with that when using the conventional TKA^28,29^. Thus, from the viewpoint of medical resources, it is difficult to perform rKA-TKA in any hospitals. To date, no study has shown the efficacy of rKA-TKA without computer-assisted surgical navigation for postoperative HKAA. Given that various rKA-TKA procedures may provide important information for surgeons and patients, we developed a manual rKA-TKA, which specializes in restricting the lateral tibial side, using a generic implant (Fig. 1, Vanguard ID; Zimmer Biomet, Warsaw, IN, USA). This implant is the only TKA design with two individual polyethylene bearings of different conformities on the medial and lateral sides. This enables surgeons to select bearings of different thicknesses and conformities personalized to patients.

We hypothesized that manual rKA-TKA can facilitate the recovery of postoperative HKAA within the safe range of ± 5°, even in patients with the preoperative HKAA ≤-19°. The current study aimed to investigate the effects of manual rKA-TKA with the modified STRT on postoperative HKAA and evaluate factors affecting postoperative HKAA in patients who underwent manual rKA-TKA. Additionally, the cut-off values of preoperative HKAA were calculated using a receiver operating characteristic (ROC) curve based on several MA-TKA-related safe zones, and postoperative HKAA was predicted using regression models.

## Results

### Patient characteristics

**Fig. 2:**
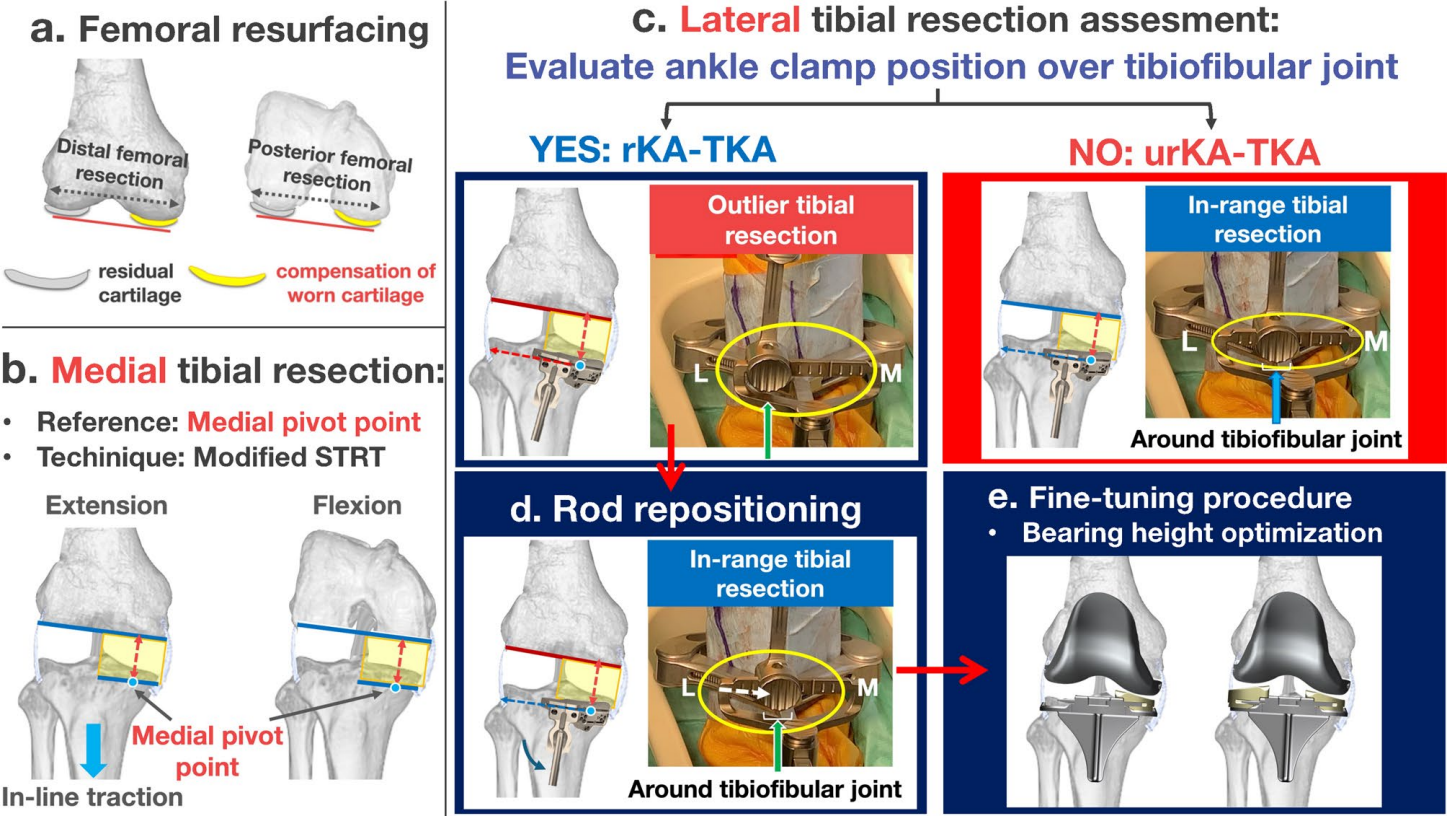
Process flowchart of the decision-making protocol for manual rKA-TKA with modified STRT. The surgical protocol consists of the following five key steps: (**a**) Femoral resurfacing procedure, (**b**) Modified STRT for determining proximal medial tibial resection, (**c**) Decision-making for rKA- or urKA-TKA based on the lateral offset position of the extramedullary tibial guide relative to the tibiofibular joint, (**d**) Execution of rKA-TKA by repositioning the offset to align with the tibiofibular joint (approximately 3° varus), (**e**) Final angular adjustment through modification of lateral bearing thickness. *rKA-TKA* restricted kinematically aligned total knee arthroplasty, *urKA-TKA* unrestricted kinematically aligned total knee arthroplasty, *STRT* soft tissue-respecting technique.

A total of 78 knees in 56 patients who underwent rKA-TKA with the modified STRT were analyzed in the current study. The median (interquartile range, IQR) age and preoperative HKAA were 77 (72–82) years and − 14° (− 17° to − 11°), respectively. The study sample included six men (approximately 11%). Tibial plate subsidence or loosening was not observed for up to 28 months after surgery. Figure 2 presents the detailed process flow for the application of rKA-TKA with the modified STRT.

### Radiographic assessment and estimated appropriate sample size

In patients (*n* = 78 knees), the average preoperative HKAA, lateral distal femoral angle (LDFA), and medial proximal tibial angle (MPTA) (standard deviation, SD) were − 14.0° (6.5°), 89.7° (2.7°), and 82.0° (3.3°), respectively, whereas the average postoperative HKAA, LDFA, and MPTA were − 3.2° (2.3°), 88.9° (1.7°), and 87.1° (2.0°), respectively. Overall, 13 knees showed severe deformity with preoperative HKAA ≤ − 19°.

**Fig. 3:**
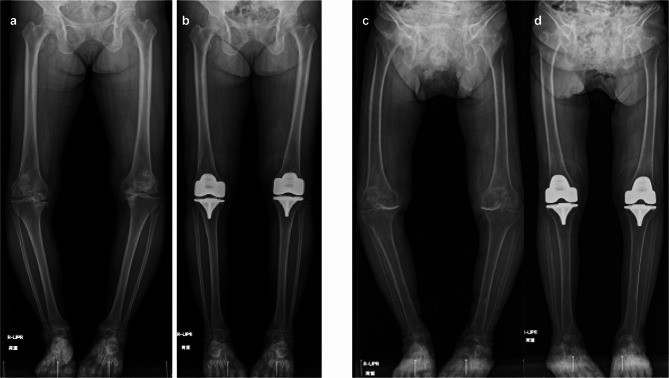
Representative pre- and postoperative radiographic findings. (**a**,**b**) Findings of a 61-year-old female. Preoperative HKAA were −21° (right) and −18° (left) varus, respectively. Postoperative HKAA are −5°(right) and −3° (left) varus, respectively. In the right knee, 9 mm of the medial bearing and 10 mm of the lateral bearing are used. In left knee, 9 mm of the medial and lateral bearing are used. (**c**,**d**) Findings of a 91-year-old female. Preoperative HKAA are −27°(right) and −22° (left) varus, respectively. Postoperative HKAA are −5°(right) and −3° (left) varus, respectively. The medial and lateral bearing of 9 mm are used in both the knees. *HKAA* hip–knee–ankle angle.

The mean and SD of the changed HKAA values at the postoperative time point were 10.9° and 5.9°, respectively. Figure 3 illustrates the findings of two representative patients showing the effects of manual rKA-TKA with the modified STRT on postoperative HKAA.

In the sample size calculation, a minimum of five patients were required for statistical significance, confirming that our sample size was sufficient for statistical analyses.

### Clinical factors affecting postoperative HKAA

Univariate and multivariate analyses were conducted to identify factors affecting postoperative HKAA (Table 1). Both analyses revealed that the preoperative HKAA was the only factor affecting postoperative HKAA (*p* < 0.001). Therefore, we used the preoperative HKAA as an explanatory variable in the subsequent analyses.

**Table 1:** Univariate and multivariate analyses of clinical factors affecting the postoperative HKAA.

	Univariate analysis		Multivariate analysis	
	Regression coefficient (95% CI)	*p*-value	Regression coefficient (95% CI)	*p*-value
Age	−3.7 (−73.3, 66.0) ×10^− 3^	0.92	0.7 (−5.7, 7.2) ×10^− 2^	0.83
Sex	−8.8 (−27.0, 9.3) ×10^− 1^	0.33	1.1 (−0.6, 2.8)	0.20
Preoperative HKAA	14.6 (7.3, 22.0) ×10^− 2^	< 0.001	14.2 (1.4, 27.0) ×10^− 2^	0.03
Preoperative LDFA	−2.8 (−4.6, −1.1) ×10^− 1^	< 0.001	−0.8 (−3.2, 1.7) ×10^− 1^	0.53
Preoperative MPTA	1.5 (0.0, 3.0) ×10^− 1^	0.06	−4.7 (−24.0, 14.7) ×10^− 2^	0.63

*CI* confidence interval, *HKAA* hip–knee–ankle angle, *LDFA* lateral distal femoral angle, *MPTA* medial proximal tibial angle.

### Cut-off values of preoperative HKAA within the safe range

By conducting an ROC curve analysis, we estimated the cut-off values of the preoperative HKAA falling within several safe ranges of the postoperative HKAA (Supplementary Table 1). The predictive ability of the preoperative HKAA was moderate (approximately 0.7 of area under the curve [AUC]) when ± 1° to ± 5° was set as the safe zone. The cut-off values of the preoperative HKAA were − 14° and − 15° when the safe zones were ± 1–3° and ± 4–5°, respectively.

### Comparison of two statistical models for estimating the postoperative HKAA

**Fig. 4:**
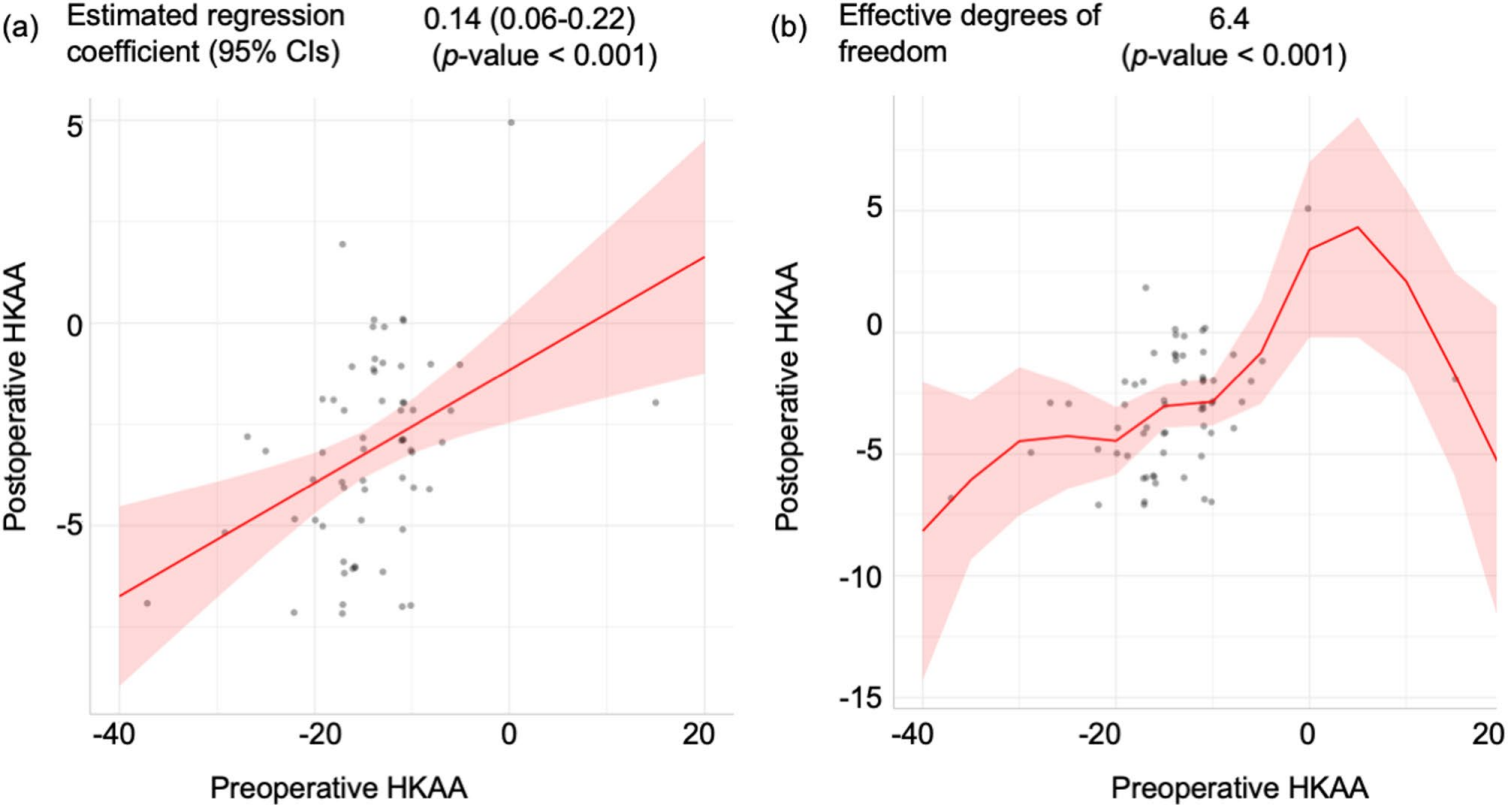
Two regression models showing the association between preoperative and postoperative HKAA. (**a**) LRM; (**b**) GAM. The regression coefficients and 95% CIs in the LRM and GAM are shown as red solid lines and red ranges, respectively. Gray circles are raw data. *CI* confidence interval, *HKAA* hip–knee–ankle angle, *GAM* generalized additive model, *LRM* linear regression model.

The surgical effects on postoperative HKAA were evaluated using the linear regression model (LRM) and generalized additive model (GAM) (Fig. 4). The LRM indicated a significant regression coefficient for the preoperative HKAA, whereas the GAM showed significant effective degrees of freedom. However, which model could more accurately predict the postoperative HKAA remained unclear. To overcome this issue, we employed a machine learning method and compared the Akaike’s information criterion (AIC) and root mean square error (RMSE) between the LRM and GAM. A model with a lower AIC and RMSE was considered to be better for predicting postoperative HKAA^30^. The AIC was 280 for the LRM and 276 for GAM, whereas the RMSE was 28.8 for the LRM and 22.5 for the GAM. These findings suggest that the GAM was a better model than the LRM in terms of predicting postoperative HKAA.

**Fig. 5:**
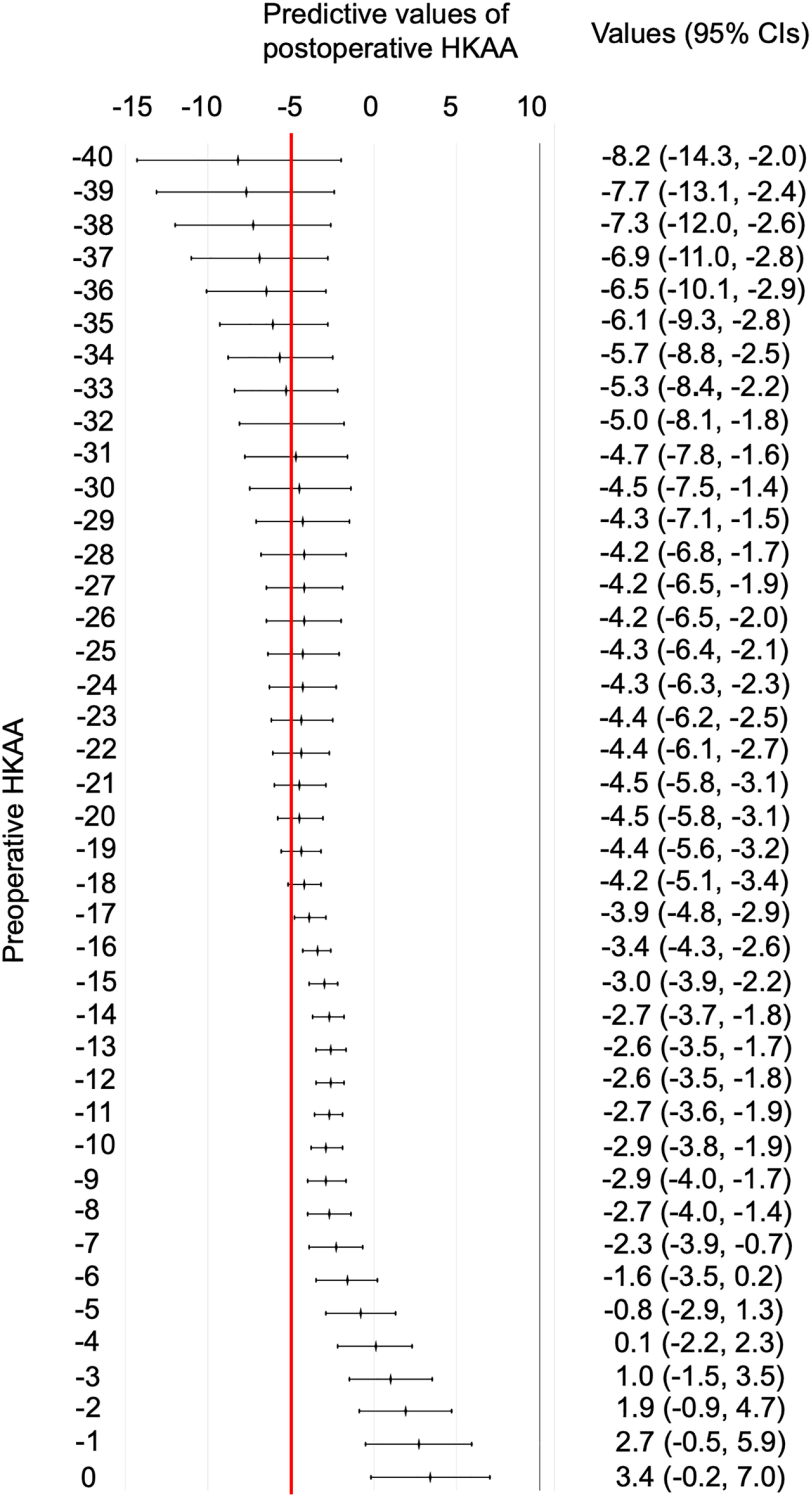
Predictive values of postoperative HKAA angle due to preoperative HKAA in the GAM. The red line indicates the postoperative HKAA of −5°. *HKAA* hip–knee–ankle angle, *GAM* generalized additive model.

Using the machine learning method with the GAM, we predicted the postoperative HKAA (Fig. 5). The 95% confidence intervals (CIs) were located right to the line of −5° of the postoperative HKAA in patients with preoperative HKAA ≥ − 17°, indicating that patients with preoperative HKAA ≥ − 17° could fall within the safe ranges. Even in patients with preoperative HKAA ≤ − 19°, the 95% CIs of postoperative HKAA included the line of − 5° of the postoperative HKAA, implying that patients with severe deformity (e.g., preoperative HKAA ≤ − 19°) could potentially fall within the MA-TKA-derived safe zone postoperatively.

## Discussion

The current study investigated the surgical effects of manual rKA-TKA with the modified STRT on postoperative HKAA. Univariate and multivariate analyses revealed that preoperative HKAA was the most relevant factor for postoperative HKAA. In the ROC curve, the optimal cut-off values of preoperative HKAA were − 14° and − 15°, respectively, when ± 1° to ± 5° was defined as the safe range of the postoperative HKAA. With respect to the association between preoperative and postoperative HKAA, the GAM was a better model to predict postoperative HKAA values, compared with the LRM (Fig. 4). The GAM predicted that even patients with severe deformity (preoperative HKAA ≤ − 19°) could potentially fall within ± 5° of the postoperative HKAA.

In urKA-TKA with the modified STRT, our previous study revealed limitations in achieving postoperative HKAA within the safe range of ± 5° for patients with severe deformity (e.g., preoperative HKAA ≤ − 19°)^21^. The rKA-TKA might overcome this issue but requires computer-assisted surgical navigation. Based on this background, we developed the manual rKA-TKA with the modified STRT and investigated its surgical effects on postoperative HKAA. The cut-off values between the ROC curve and the GAM were different. Although it remains debatable which cut-off values can be adopted clinically, we recommend the use of the latter for the following reasons: (i) the ROC curve analyses lacked the ability to predict the actual postoperative HKAA values; and (ii) there exists no consensus on the safe range of KA-TKA in postoperative HKAA^20^. The GAM findings, as shown in Fig. 5, indicate that our current surgical approach can overcome the limitations of urKA-TKA with the modified STRT.

**Fig. 6:**
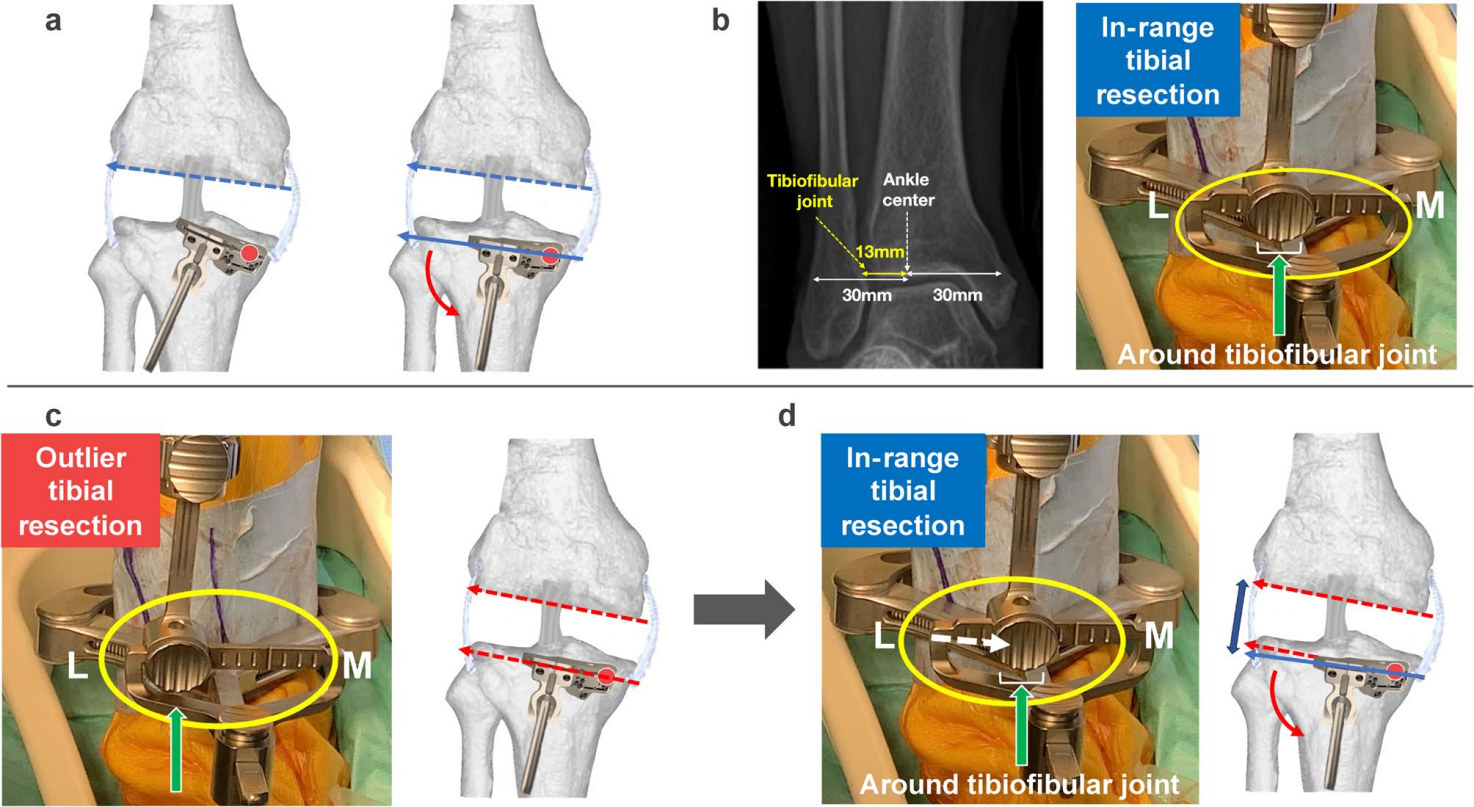
Surgical steps of tibial resection in manual rKA-TKA (**a**–**c**). (**a**) Confirming the lateral tibial resection level with a fixed medial pivot point (red circle) using the modified STRT. The lateral tibial resection (right: blue solid arrow) is made parallel to the distal femur (right: blue dotted arrow) in extension. (**b**) Evaluation of urKA or rKA-TKA procedures. The tibiofibular joint location is determined by measuring the distance from the ankle center to the tibiofibular joint on preoperative radiographs (left: preoperative measurement). When the ankle clamp offset aligns around the tibiofibular joint (right: green solid arrow) (approximately 13 mm lateral offset from ankle center in this image), this indicates an in-range tibial cut suitable for urKA-TKA. (**c**) Application of rKA-TKA restrictions to the lateral tibia. When lateral offset is observed in the ankle clamp after modified STRT, realignment around the tibiofibular joint is required. This scenario represents an outlier tibial resection due to severe varus tibial resection angle (right: red dotted arrow on tibia). (**d**) Tibial resection technique in rKA-TKA. After repositioning the offset (left: white dotted arrow), the tibiofibular joint is defined as the in-range reference area (left: green solid arrow) while maintaining the fixed medial pivot point. The volume of lateral resection in the rKA (right: blue solid arrow on the tibia) is slightly larger than that in the urKA (right: red dotted arrow on the tibia). *rKA-TKA* restricted kinematically aligned total knee arthroplasty, *urKA-TKA* unrestricted kinematically aligned total knee arthroplasty, *STRT* soft tissue-respecting technique.

**Fig. 7:**
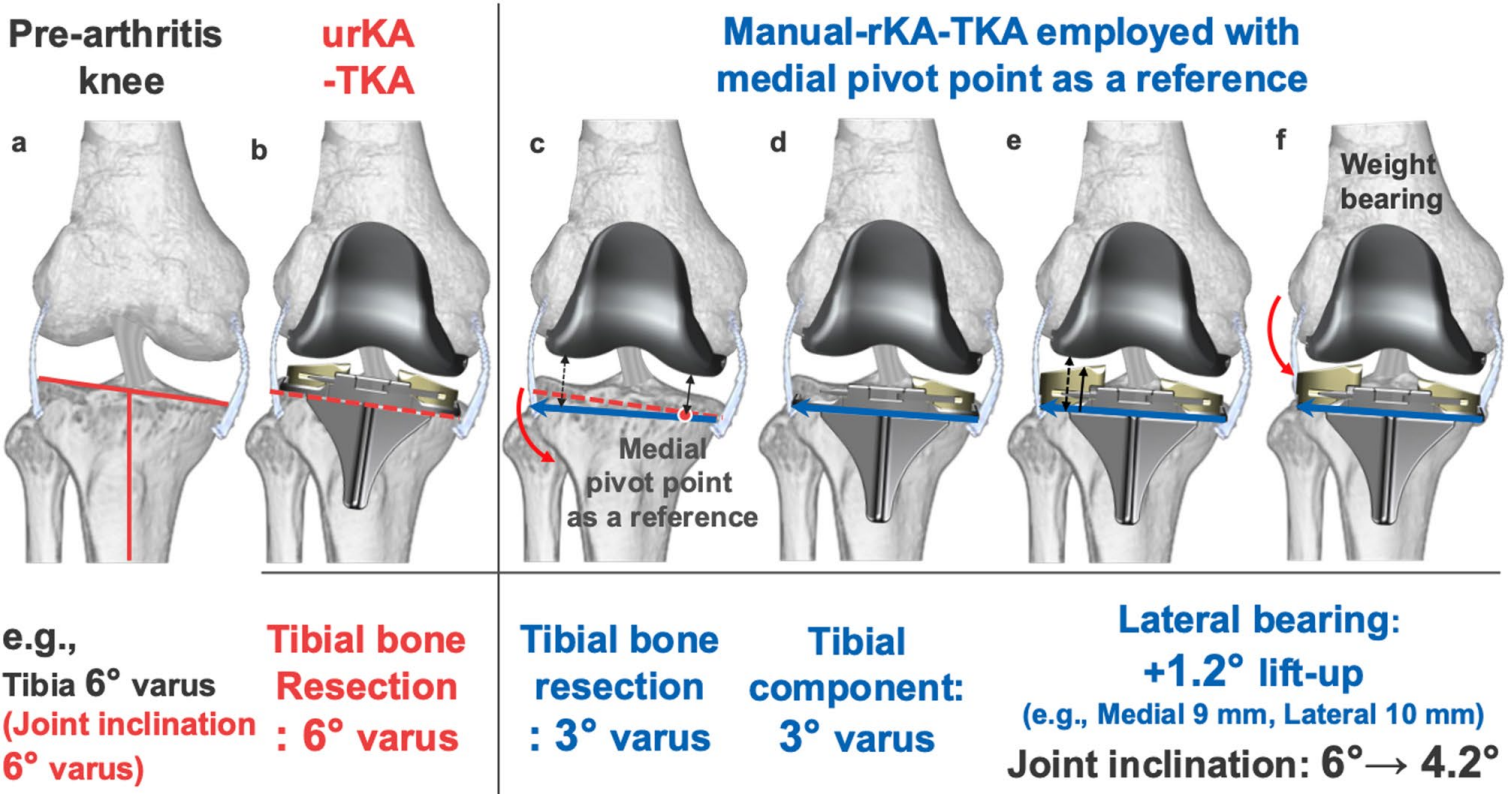
The concept of manual rKA-TKA with the modified STRT: (**a**) Pre-arthritic knee showing 6° tibial varus angle. (**b**) 6° tibial varus resection angle following urKA-TKA with caliper technique. (**c**) Using modified STRT, the medial tibial resection level is verified in both extension and flexion (employing the medial pivot point: red circle). In the rKA-TKA, the tibial resection is made at 3° varus while maintaining a fixed medial pivot point (blue solid arrow). This creates a larger lateral space (black bidirectional dotted arrow) compared to the medial space (black bidirectional solid arrow). (**d**) The final angular adjustment is achieved by modifying the lateral bearing thickness. Initially, the tibial trial component is aligned with a 3° varus. The appropriate medial bearing thickness is selected after assessing the medial gap between femoral and tibial trial components. (**e**) Subsequently, lateral bearing thickness (black solid arrow) is selected to be less than the available lateral space (black bidirectional dotted arrow). For example, the lateral bearing is elevated only 1 mm relative to the medial bearing (e.g., medial bearing 9 mm, lateral bearing 10 mm: resulting in 1.2° joint inclination), rather than 2 mm (e.g., medial bearing 9 mm, lateral bearing 11 mm: resulting in 2.4° joint inclination). (**f**) This reduces the joint inclination from 6 to 4.2°, potentially decreasing the postoperative HKAA varus angle by 1.8° on whole-leg weight-bearing radiographs. In summary, when applying the rKA-TKA, the lateral tibia is cut slightly more than the component thickness and adjusted with the lateral bearing to reduce the postoperative HKAA. Therefore, a slight lateral slack in extension under non-weight bearing is acceptable. *HKAA* hip–knee–ankle angle, *rKA-TKA* restricted kinematically aligned total knee arthroplasty, *urKA-TKA* unrestricted kinematically aligned total knee arthroplasty, *STRT* soft tissue-respecting technique.

Theoretically, in urKA-TKA using the caliper technique, tibial resection is independent of soft tissue, and the postoperative HKAA depends on the preoperative HKAA. In contrast, urKA-TKA with STRT is regarded as a soft tissue-dependent tibial resection procedure, although the femoral resurfacing concept is the same as that of the calipers^11,13,21^. In STRT, the medial joint space can become slightly wider after resecting the medial osteophytes, which affects the preoperative HKAA in a varus knee^31,32^. Under these conditions, the tibial bone cutting plane was determined to be parallel to the distal femur during the manual in-line traction technique. Therefore, compared with the caliper technique, STRT itself has some potential surgical effects on postoperative HKAA. Our previous study revealed that urKA-TKA with the modified-STRT can possess the potential to restore the postoperative HKAA to fall within − 5° even in patients with the preoperative HKAA ≥ − 18°^21^. Based on these findings, we hypothesized that angular restriction to fall within the safe zone might be needed by only a few degrees, and angular restriction might be sufficient only on the tibial side after femoral resurfacing in the application of STRT. Therefore, we developed a technique using the Vanguard ID to manually control the angular restriction on the lateral tibial side (Figs. 1, 2 and 6). Our surgical approach follows the principle of “not perfect, but good enough,” meaning that slight variations in bone resection angles are acceptable within a defined range. The key to our procedure lies in establishing the medial pivot point on the tibia, which guides the appropriate level of medial tibial resection using a medial-stabilizing technique (Fig. 2b). As reproducing medial stability is important for improving post-TKA functional outcomes^33^, determination of the medial pivot point in rKA-TKA allows medial stability in the entire range of motion to restore the positional relationship between the pre-arthritic joint line and medial collateral ligament. Regarding the concept, the final angular adjustment is performed by changing the thickness of the lateral bearing after rough lateral tibial resection (Figs. 2 and 7). Specifically, when rKA-TKA is performed, the lateral tibia cutting thickness can be greater than the implant thickness. Subsequently, the thickness of the lateral bearing was raised to less than the lateral implant space to avoid excessive residual postoperative HKAA (accepting for slight loosening of the lateral space in extension) (Figs. 2 and 7). Manual angular adjustment after rough tibial resection allows precise angular adjustment without computer-assisted surgical navigation.

Manual rKA-TKA with the modified STRT developed in the current study is advantageous. First, it allows surgeons to perform rKA-TKA without computer-assisted surgical navigation. This may contribute to the generalizability of rKA-TKA. Second, a precise tibial resection angle is not required. The conventional cutting guide lacks the required precise resection because 30% of knees can possess a risk of greater than 3° malalignment^34,35^. In our method, the medial pivot point was fixed, and the amount of lateral tibial resection was slightly greater than the component thickness (Figs. 2, 6 and 7). Subsequently, the lateral component space was adjusted based on the thickness of the lateral bearing. Thus, our method allows imprecise bone resection using a conventional cutting guide. The third advantage was the excellent surgical effects even in patients with the postoperative HKAA ≤ − 19° (Fig. 5). As urKA-TKA with the modified STRT can be less likely to fall within − 5° of the postoperative HKAA, these patients might be the appropriate for the application of the manual rKA-TKA. However, surgeons should be aware of the disadvantages of the manual rKA-TKA. The first disadvantage is that the HKAA cannot be quantitatively restricted because of manual determination using a curved gap gauge in the final lateral component space adjustment. Surgeons’ experience with our surgical methods can affect the degree of adjustment among surgeons, or within-surgeon variability in experience might affect postoperative HKAA. The second disadvantage is the dependence on visual inspection of the location of the lateral offset in the ankle clamp of the extramedullary tibial guide, although preoperative radiographic measurements were used as a reference. Visual inspection can be an inaccurate indicator, increasing the variability in the surgical effects on postoperative HKAA. As this surgical method performs only angular restriction to the tibial bone, some surgeons might regard this method’s low angular limitation as a third disadvantage. However, STRT itself appears to influence postoperative HKAA in urKA-TKA^21^. Based on our findings, few patients would require additional angular restrictions.

Regarding the femur, full-thickness cartilage loss in the distal femur is generally approximately 2 mm; therefore, surgeons usually compensate for 1–2 mm cartilage loss to restore the femoral distal articular surface in urKA-TKA. In contrast, the rKA-TKA algorithm, which is mainly based on the usage of computer-assisted surgical navigation, consists of adjusting the LDFA and MPTA for patients with severe degrees of preoperative HKAA^22^. However, there is no strategy to compensate for the distal femur to restore the near-normal pre-arthritic articular surface in manual rKA-TKA. In clinical practice, there are many patients of cartilage loss and bone defects of the distal femur in patients with severe degrees of preoperative HKAA, especially in Asia. Thus, to achieve restoring the near-normal pre-arthritic articular surface of femur, we attempted to compensate 3–4 mm of cartilage and bone loss in distal femur in patients of severe varus femur (LDFA > 93°). However, in this study, the degree of preoperative LDFA was not associated with postoperative HKAA in the multivariate analysis. However, we found that patients with the severe deformity in the preoperative HKAA ≤ − 19° can possess the potential of falling within ± 5° of the postoperative HKAA using the manual-rKA-TKA (Fig. 5). Generally, patients of severe preoperative HKAA include severe LDFA and MPTA. Therefore, we assumed that the compensation in patients with severely varus femurs (e.g., LDFA > 93°) may allow the excellent results.

Our study had some limitations. First, this study included a lower number of patients with preoperative HKAA ≤ − 19° (*n* = 13). The 95% CIs of estimated postoperative HKAA ≤ − 20° in the GAM were wider than those of estimated postoperative HKAA ≥ − 19° and ≤ − 10° (Figs. 4 and 5). When predicting the postoperative HKAA in patients with preoperative HKAA ≤ − 19° for the application of our surgical methods, surgeons should understand the wider range of the predicted values provided by the GAM. Second, this was a retrospective study with a single-group design. All surgeries were performed by a single surgeon (primary operator), making it impossible to investigate the effects of surgeon variability or within-surgeon variability in surgical experience on postoperative HKAA.

In conclusion, we established manual rKA-TKA with the modified STRT and investigated its surgical effects on postoperative HKAA. The most significant advantage of this surgical method is the possibility of performing rKA-TKA without computer-assisted surgical navigation. Furthermore, excellent surgical effects on postoperative HKAA can be expected, even in patients with severe deformity of preoperative HKAA. Therefore, for surgeons working at resource-limited institutions performing TKA in patients with preoperative HKAA ≤ − 19°, manual rKA-TKA may serve as a viable surgical option. Our surgical methods may be helpful in achieving the generalizability of rKA-TKA and may contribute to the provision of novel insights into surgical indications rKA-TKA.

## Methods

### Patient selection

The Research Ethics Committee of Nagahama Red Cross Hospital approved the study protocol (approval no. 2023-002). All experiments were performed in accordance with the Declaration of Helsinki. Written informed consent was obtained from all participants. The requirement for informed consent from patients was waived owing to the retrospective nature of this study.

Patients who underwent rKA-TKA with the modified STRT using posterior cruciate ligament-retaining implants (Vanguard ID; Zimmer Biomet, Warsaw, IN, USA) at our institution from January 2021 to January 2024 were retrospectively reviewed. Patients diagnosed with knee osteoarthritis were included in this study. However, patients with collateral knee ligament injury, history of femoral/tibial osteotomy, or trauma around the knee were excluded.

### Assessment

Age, sex, and preoperative HKAA were collected as explanatory variables for statistical analysis. Weight-bearing full-length anteroposterior radiographs were obtained before and after surgery. Additionally, the mechanical HKAA, LDFA, and MPTA were assessed.

### Implant characteristics

Vanguard ID is the unique implant capable of individually customizing the medial and lateral polyethylene bearings through differential thickness and constraints (Fig. 1).

#### Thickness

Vanguard ID allows for changes in thickness independently. Every 1-mm difference in the bearing results in 1.2° of joint-line inclination.

#### Constraints

Vanguard ID also allows for changes in conformity independently. Two types of bearing conformity exist—namely, the anterior stabilized (AS) bearing with high constrained design and the bicruciate-preserving (XP) bearing with low constrained design. In this study, an AS bearing was used on the medial side, whereas an XP bearing was utilized on the lateral side.

### Surgical procedures

A single primary surgeon (first author, M.I.) performed manual rKA-TKA and made all decisions regarding surgical procedures. As previously described, all knee joints were exposed using the modified subvastus approach and modified STRT^21^. The distance from the ankle joint center to the tibiofibular joint was measured on a preoperative full-length radiograph (Fig. 6b).

#### Distal and posterior femoral resection: resurfacing the femur

Based on the kinematic alignment principle, the femur in each patient was resurfaced, as described previously^11,12,36^. Briefly, following compensation for the extent of cartilage loss (1–2 mm), the distal and posterior femur was cut to a thickness equivalent to that of the femoral component. Furthermore, in the current surgical method, we attempted to perform a 3–4-mm compensation of the distal femur, depending on intraoperative findings, for patients with a severe varus femur (LDFA > 93°) from the viewpoint of the presence of cartilage and bone loss.

#### Determination of the proximal tibial resection level: modified STRT with the medial pivot point as a reference

After femoral resurfacing, the medial tibial resection level in extension (medial pivot point in extension) was confirmed using the manual in-line traction technique (original STRT)^17,21^. Subsequently, the medial tibial resection level in flexion (medial pivot point in flexion) was confirmed using an Oxford extramural tibial saw guide. If the medial pivot point was inconsistent with extension and flexion, an intermediate height was defined as the level of medial resection. This double-checking method (modified STRT) allowed for the reproduction of near-pre-arthritic condition relationships between the medial side of the distal femur and proximal tibia (medial stabilizing technique); thus, the level of medial tibial resection (medial pivot point) could be fixed in all patients. The level of lateral tibial resection was determined to be parallel to the distal femur using the manual in-line traction technique.

#### Application of manual rKA-TKA with lateral tibial side resection

The application of rKA-TKA was determined based on the lateral offset location in the ankle clamp of the extramedullary tibial resection guide and the lateral tibial resection thickness.

When determining the lateral tibial resection level, patients were classified into two groups: the “in-range” group, wherein urKA-TKA was performed when the offset was located around the tibiofibular joint (referred to as preoperative measurements) (Fig. 6a, b), and the “outlier” group, wherein rKA-TKA was used when the offset exceeded the tibiofibular joint (Fig. 6c). In the outlier group, the offset was adjusted back to around the tibiofibular joint to restrict the tibial resection angle (Fig. 6d).

In rKA-TKA procedures, the lateral tibial resection thickness typically exceeded the component thickness. For example, in varus knees, while the tibial component thickness was 9 mm, the lateral tibial resection thickness was typically 11–12 mm.

#### Fine-tuning procedure of lateral component space using the distinctive characteristics of vanguard ID

Taking advantage of Vanguard ID to change the thickness of each bearing, final angular adjustments in rKA-TKA were made to modify bearing thicknesses (Figs. 2 and 7). During the component trial, the component space was measured using a curved gap gauge. Subsequently, the medial bearing thickness was selected to achieve approximately 1.5 mm of medial component space. The lateral bearing thickness was then determined to be slightly lesser than that of the lateral component space to prevent excessive residual postoperative HKAA.

### Data analysis

Age and preoperative HKAA, LDFA, and MPTA were continuous variables, whereas sex was a dichotomous variable. Quantitative variables are presented as means or medians with IQRs or 95% CIs. The sample size required for the study was calculated using a paired t-test to confirm whether the sample size in our data was statistically sufficient. The means and SDs of the changed HKAA values at the postoperative time point were utilized to calculate the sample size, with a power of 90% and two-sided alpha level of 0.05. Univariate and multivariate analyses were conducted to investigate factors influencing postoperative HKAA, with age, sex, and preoperative HKAA/LDFA/MPTA as the explanatory variables and with postoperative HKAA as the response variable. The LRM was used for multivariate analysis.

As no consensus on the safe range of postoperative HKAA in KA-TKA has yet been reached^20^, we assumed several safe ranges and determined the AUC using the ROC curve. Furthermore, we calculated the respective cut-off values of preoperative HKAA within each safe range postoperatively and determined the optimal cut-off values using the Youden’s index.

Previously, we reported that a nonlinear regression model could more accurately explain the association between preoperative and postoperative HKAA than a linear regression model^21^. Therefore, the LRM, which represents the linear association between the response and explanatory variables, and the GAM, which represents the nonlinear association between the response and explanatory variables, were used in our study. With these models, we predicted the postoperative HKAA based on the preoperative values in rKA-TKA with the modified STRT. As the use of all data for the analysis results in overfitting, we employed machine learning to avoid this issue and determined a better model for predicting postoperative HKAA. We used the “modelr” package in R (The R Foundation for Statistical Computing, Vienna, Austria) and subsequently divided the data into training and test data under a 10-fold cross-validation. We utilized the training dataset and introduced the explanatory variables most relevant to postoperative HKAA into the LRM and GAM. Then, we calculated the AIC. Additionally, the RMSE was calculated using the test dataset.

All statistical analyses were performed using R software version 3.6.1 (The R Foundation for Statistical Computing), with statistical significance set at a level of 5%.

## Supplementary Information

Below is the link to the electronic supplementary material.

Supplementary Material 1

## Data Availability

The data supporting the findings of this study are available upon request from the corresponding author.

## References

[1] Font-Rodriguez, D. E., Scuderi, G. R. & Insall, J. N. Survivorship of cemented total knee arthroplasty. *Clin. Orthop. Relat. Res.* **345**, 79–86 (1997). 9418624

[2] Insall, J. N., Hood, R. W., Flawn, L. B. & Sullivan, D. J. The total condylar knee prosthesis in gonarthrosis. A five to nine-year follow-up of the first one hundred consecutive replacements. *J. Bone Joint Surg. Am.* **65**, 619–628 (1983). 6853567

[3] Collier, M. B., Engh, C. A., McAuley, J. P. & Engh, G. A. Factors associated with the loss of thickness of polyethylene tibial bearings after knee arthroplasty. *J. Bone Joint Surg. Am.* **89**, 1306–1314 (2007). 17545435 10.2106/JBJS.F.00667

[4] Jeffery, R. S., Morris, R. W. & Denham, R. A. Coronal alignment after total knee replacement. *J. Bone Joint Surg. Br.* **73**, 709–714 (1991). 1894655 10.1302/0301-620X.73B5.1894655

[5] D’Lima, D. D., Chen, P. C. & Colwell, C. W. Polyethylene contact stresses, articular congruity, and knee alignment. *Clin. Orthop. Relat. Res.* **392**, 232–238 (2001). 10.1097/00003086-200111000-00029 11716388

[6] Bourne, R. B., Chesworth, B. M., Davis, A. M., Mahomed, N. N. & Charron, K. D. J. Patient satisfaction after total knee arthroplasty: who is satisfied and who is not? *Clin. Orthop. Relat. Res.* **468**, 57–63 (2010). 19844772 10.1007/s11999-009-1119-9 PMC2795819

[7] Baker, P. N., van der Meulen, J. H., Lewsey, J. & Gregg, P. J. National joint registry for England and wales. The role of pain and function in determining patient satisfaction after total knee replacement. Data from the National joint registry for England and Wales. *J. Bone Joint Surg. Br.* **89**, 893–900 (2007). 17673581 10.1302/0301-620X.89B7.19091

[8] Bellemans, J., Colyn, W., Vandenneucker, H. & Victor, J. The Chitranjan Ranawat award: is neutral mechanical alignment normal for all patients? The concept of constitutional varus. *Clin. Orthop. Relat. Res.* **470**, 45–53 (2012). 21656315 10.1007/s11999-011-1936-5 PMC3237976

[9] Butarbutar, J. C. P., Mandagi, T., Siahaan, L. D., Suginawan, E. T. & Elson, I. Prevalence of proximal tibia vara in Indonesian population with knee osteoarthritis. *J. Clin. Orthop. Trauma.* **29**, 101871 (2022). 35510147 10.1016/j.jcot.2022.101871 PMC9058951

[10] Shetty, G. M., Mullaji, A., Bhayde, S., Nha, K. W. & Oh, H. K. Factors contributing to inherent varus alignment of lower limb in normal Asian adults: role of tibial plateau inclination. *Knee* **21**, 544–548 (2014). 24139813 10.1016/j.knee.2013.09.008

[11] Howell, S. M., Papadopoulos, S., Kuznik, K. T. & Hull, M. L. Accurate alignment and high function after kinematically aligned TKA performed with generic instruments. *Knee Surg. Sports Traumatol. Arthrosc.* **21**, 2271–2280 (2013). 23948721 10.1007/s00167-013-2621-x

[12] Howell, S. M., Papadopoulos, S., Kuznik, K., Ghaly, L. R. & Hull, M. L. Does varus alignment adversely affect implant survival and function six years after kinematically aligned total knee arthroplasty? *Int. Orthop.* **39**, 2117–2124 (2015). 25823516 10.1007/s00264-015-2743-5

[13] Rivière, C. et al. Alignment options for total knee arthroplasty: a systematic review. *Orthop. Traumatol. Surg. Res.* **103**, 1047–1056 (2017). 28864235 10.1016/j.otsr.2017.07.010

[14] Morcos, M. W., Uhuebor, D. & Vendittoli, P. A. Overview of the different personalized total knee arthroplasty with robotic assistance, how choosing? *Front. Surg.* **10**, 1120908 (2023). 36936647 10.3389/fsurg.2023.1120908 PMC10020354

[15] Eckhoff, D. G. et al. Three-dimensional mechanics, kinematics, and morphology of the knee viewed in virtual reality. *J. Bone Joint Surg. Am.* **87** (Suppl 2), 71–80 (2005). 16326726 10.2106/JBJS.E.00440

[16] Hiranaka, T. et al. Current concept of kinematic alignment total knee arthroplasty and its derivatives. *Bone Jt. Open.* **3**, 390–397 (2022). 35532356 10.1302/2633-1462.35.BJO-2022-0021.R2 PMC9134837

[17] Soda, Y., Nakamura, M. & Adachi, N. Coronal alignment of three different types of implants in kinematically aligned total knee arthroplasty: a comparative study. *Open. J. Orthop.* **11**, 183–198. 10.4236/ojo.2021.116018 (2021).

[18] Huber, K., Christen, B., Calliess, S. & Calliess, T. True kinematic alignment is applicable in 44% of patients applying restrictive indication criteria-a retrospective analysis of 111 TKA using robotic assistance. *J. Pers. Med.* **11**, 662 (2021). 34357129 10.3390/jpm11070662 PMC8307604

[19] Suda, Y. et al. Approximately 80% of Japanese Osteoarthritic patients fall out of the safety range in restricted kinematically-aligned total knee arthroplasty in an analysis of preoperative long-leg radiograms. *Knee* **35**, 54–60 (2022). 35220133 10.1016/j.knee.2022.02.008

[20] Schelker, B. L., Nowakowski, A. M. & Hirschmann, M. T. What is the safe zone for transition of coronal alignment from systematic to a more personalised one in total knee arthroplasty? A systematic review. *Knee Surg. Sports Traumatol. Arthrosc.* **30**, 419–427 (2022). 34973095 10.1007/s00167-021-06811-5 PMC8866271

[21] Ishikawa, M. et al. Effects of unrestricted kinematically aligned total knee arthroplasty with a modified soft-tissue respecting technique on the deformity of limb alignment in Japanese patients. *Medicina (Kaunas)* **59**, (2023). 10.3390/medicina59111969 PMC10673030 38004019

[22] Vendittoli, P. A., Martinov, S. & Blakeney, W. G. Restricted kinematic alignment, the fundamentals, and clinical applications. *Front. Surg.* **8**, 697020 (2021). 34355018 10.3389/fsurg.2021.697020 PMC8329359

[23] Almaawi, A. M., Hutt, J. R. B., Masse, V., Lavigne, M. & Vendittoli, P. A. The impact of mechanical and restricted kinematic alignment on knee anatomy in total knee arthroplasty. *J. Arthroplasty* **32**, 2133–2140 (2017). 28302462 10.1016/j.arth.2017.02.028

[24] MacDessi, S. J. et al. Restoring the constitutional alignment with a restrictive kinematic protocol improves quantitative soft-tissue balance in total knee arthroplasty: a randomized controlled trial. *Bone Joint J.* **102–B**, 117–124 (2020). 10.1302/0301-620X.102B1.BJJ-2019-0674.R2 PMC6974544 31888372

[25] Risitano, S. et al. Restricted kinematic alignment in primary total knee arthroplasty: a systematic review of radiographic and clinical data. *J. Orthop.* **33**, 37–43 (2022). 35812351 10.1016/j.jor.2022.06.014 PMC9263746

[26] Massé, V., Cholewa, J. & Shahin, M. Personalized alignment™ for total knee arthroplasty using the Rosa^®^ knee and Persona^®^ knee systems: surgical technique. *Front. Surg.* **9**, 1098504 (2022). 36733674 10.3389/fsurg.2022.1098504 PMC9888495

[27] MacDessi, S. J. Restricted kinematic alignment in total knee arthroplasty: scientific exploration involving detailed planning, precise execution, and knowledge of when to abort. *Arthroplast Today*. **10**, 24–26 (2021). 34277907 10.1016/j.artd.2021.05.024 PMC8267482

[28] Tompkins, G. S., Sypher, K. S., Li, H. F., Griffin, T. M. & Duwelius, P. J. Robotic versus manual total knee arthroplasty in high volume surgeons: A comparison of cost and quality metrics. *J. Arthroplasty*. **37**, S782–S789 (2022). 34952162 10.1016/j.arth.2021.12.018

[29] Christen, B. et al. Comparative cost analysis of four different computer-assisted technologies to implant a total knee arthroplasty over conventional instrumentation. *J. Pers. Med.* **12** (2022). 10.3390/jpm12020184 PMC8880057 35207672

[30] Harrison, X. A. et al. A brief introduction to mixed effects modelling and multi-model inference in ecology. *PeerJ* **6**, e4794 (2018). 29844961 10.7717/peerj.4794 PMC5970551

[31] Nishihara, N. et al. Correction of varus alignment with peripheral osteophyte removal during total knee arthroplasty: an assessment with computer navigation. *J. Knee Surg.* **36**, 292–297 (2023). 34520563 10.1055/s-0041-1731737

[32] Mullaji, A. Can isolated removal of osteophytes achieve correction of varus deformity and gap-balance in computer-assisted total knee arthroplasty? *Bone Joint J.* **102–B**, 49–58 (2020). 10.1302/0301-620X.102B6.BJJ-2019-1597.R1 32475289

[33] Matsuda, S. & Ito, H. Ligament balancing in total knee arthroplasty – medial stabilizing technique. *Asia Pac. J. Sports Med. Arthrosc. Rehabil Technol.* **2**, 108–113 (2015). 29264249 10.1016/j.asmart.2015.07.002 PMC5730662

[34] Mason, J. B., Fehring, T. K., Estok, R., Banel, D. & Fahrbach, K. Meta-analysis of alignment outcomes in computer-assisted total knee arthroplasty surgery. *J. Arthroplasty*. **22**, 1097–1106 (2007). 18078876 10.1016/j.arth.2007.08.001

[35] Hetaimish, B. M. et al. Meta-analysis of navigation vs conventional total knee arthroplasty. *J. Arthroplasty*. **27**, 1177–1182 (2012). 22333865 10.1016/j.arth.2011.12.028

[36] Rivière, C., Villet, L., Jeremic, D. & Vendittoli, P. A. What you need to know about kinematic alignment for total knee arthroplasty. *Orthop. Traumatol. Surg. Res.* **107**, 102773 (2021). 33333274 10.1016/j.otsr.2020.102773

