# Supplementary material for: Manual restricted kinematic alignment technique restores postoperative limb alignment in severe knee deformities
Source: Sci Rep. 2025 Jul 30;15:27757. doi: 10.1038/s41598-025-13195-w (PMC12311097; doi:10.1038/s41598-025-13195-w)
Supplement: Supplementary file 1 — Supplementary Material 1 [file 41598_2025_13195_MOESM1_ESM.docx]

| **Supplementary table 1.** Result of ROC curve for deciding the cut-off values of the preoperative HKAA based on different safe ranges of the postoperative HKAA. | | | | |
| --- | --- | --- | --- | --- |
| **Safe range of the postoperative HKAA** | **AUC**  **(95% CI)** | **Sensitivity** | **Specificity** | **Cut-off values of preoperative HKAA** |
| ±1 | 0.70  (0.57, 0.83) | 0.88 | 0.58 | -14 |
| ±2 | 0.72  (0.60, 0.84) | 0.82 | 0.65 | -14 |
| ±3 | 0.70  (0.58, 0.82) | 0.71 | 0.69 | -14 |
| ±4 | 0.73  (0.60, 0.85) | 0.60 | 0.85 | -15 |
| ±5 | 0.67  (0.53, 0.82) | 0.67 | 0.77 | -15 |
| Abbreviations: AUC, area under the curve; CI, confidence interval; HKAA, hip–knee–ankle angle; ROC, receiver operating characteristic. | | | | |
